# A Real-Time Digital Twin Synchronization Framework for Multi-Sensor Cardiopulmonary Resuscitation Measurement

**DOI:** 10.3390/s26113459

**Published:** 2026-05-30

**Authors:** Kai-Chao Yao, Feng-Yu Lin, Sumei Chiang

**Affiliations:** Department of Electrical and Mechanical Technology, College of Technology, National Changhua University of Education, Bao-Shan Campus, No. 2, Shi-Da Rd., Changhua City 500208, Taiwan; kcyao@cc.ncue.edu.tw (K.-C.Y.); d1331008@mail.ncue.edu.tw (F.-Y.L.)

**Keywords:** digital twin, CPR compression measurement, multi-sensor integration, IoT sensing, LabVIEW, real-time feedback

## Abstract

This study proposes a digital twin-based CPR compression measurement system (DTCMS) architecture for real-time monitoring of CPR compression. The system combines a load cell, an inertial measurement unit (IMU), a LabVIEW acquisition platform, and a CNN module to capture multi-modal motion characteristics during CPR repetitive compression training. A calibration-aware sensor fusion framework synchronizes heterogeneous signals, reduces drift, and enhances robustness under high-frequency operation. Real-time data acquisition, latency-controlled transmission, and digital twin visualization enable synchronized physical–virtual interaction. Experimental results demonstrate high accuracy (*R*^2^ > 0.99), stable repeatability (coefficient of variation: CV < 3.5%), and reliable dynamic tracking. The compression depth error was maintained within ±1.5 mm, and synchronization latency remained below 0.2 s. Results confirm the proposed DTCMS architecture as a robust solution for real-time biomechanical monitoring and digital twin-based interactive systems. Compared with conventional single-sensor CPR monitoring systems, the proposed framework improves synchronization stability and sensing robustness through calibration-aware multi-sensor fusion.

## 1. Introduction

Cardiopulmonary resuscitation (CPR) is a critical life-saving skill in which compression depth and frequency directly affect patient survival and neurological outcomes [1]. According to the American Heart Association (AHA) guidelines, chest compressions should reach 5–6 cm in depth at a rate of 100–120 per minute, with full recoil [2]. However, conventional training devices typically rely on single-axis pressure sensors or mechanical feedback, which provide limited accuracy and lack objective, real-time data. These limitations hinder precise training and systematic evaluation during CPR practice [3].

Recent advances in IoT, artificial intelligence (AI), and digital twin (DT) technologies have enabled cyber–physical synchronization and intelligent medical training systems [4]. While DT has been widely applied in manufacturing and surgical simulation, its adoption in CPR training remains at an early stage. Existing CPR training systems fail to integrate multi-sensor data acquisition, real-time calibration, and synchronized virtual mapping [5]. To bridge this gap, we propose a digital twin-based CPR compression measurement system (DTCMS) that integrates a load cell, IMU, LabVIEW acquisition platform, and a CNN module. This system enables high-precision compression depth detection, posture monitoring, and real-time synchronization with a virtual model. In this study, the term “digital twin” primarily refers to a real-time cyber–physical synchronization framework that maintains continuous temporal consistency between physical CPR operations and their corresponding virtual representations. Unlike advanced digital twin systems that incorporate predictive simulation, autonomous decision-making, or adaptive control, the proposed architecture focuses on synchronized sensing, real-time visualization, and low-latency data interaction for CPR measurement applications. Therefore, the proposed DTCMS should be interpreted as a synchronization-oriented digital twin prototype designed for real-time biomechanical sensing and visualization rather than a fully autonomous intelligent digital twin platform.

The primary novelty of this study does not lie in the independent use of load cells, IMUs, LabVIEW, or CNN techniques, which have been individually investigated in prior studies. Instead, the contribution of this work is the development of a calibration-aware multi-sensor synchronization framework for real-time CPR digital twin sensing. The proposed architecture integrates heterogeneous sensing modalities through timestamp-aligned sensor fusion, indirect force–displacement estimation, and low-latency cyber–physical synchronization. Unlike conventional CPR monitoring systems that primarily rely on single-sensor feedback or isolated visualization modules, the proposed DTCMS establishes a unified sensing and synchronization pipeline capable of maintaining stable measurement consistency under repetitive dynamic compression conditions. The major contributions of this study are summarized as follows:

(1) A calibration-aware multi-sensor sensing framework integrating load-cell and IMU measurements for synchronized CPR biomechanical monitoring.

(2) A real-time cyber–physical synchronization architecture enabling low-latency digital twin mapping with stable temporal alignment below 0.2 s.

(3) An indirect displacement estimation mechanism based on calibrated force–displacement conversion, reducing hardware complexity while maintaining high sensing precision.

(4) A synchronization-oriented sensor fusion strategy designed to improve robustness against motion instability and posture-induced sensing deviation during repetitive CPR operations.

(5) Experimental validation demonstrating high linearity, repeatability, and dynamic sensing stability suitable for real-time medical training environments.

## 2. Literature Review

### 2.1. CPR Measurement Devices

In CPR training, the measurement accuracy and real-time performance of simulation devices are crucial to learning effectiveness [6]. Currently, most traditional pressure-sensing CPR simulators use a single pressure sensor. However, smart CPR systems have emerged in recent years. These simulators, which integrate Bluetooth transmission and mobile device applications, cannot accurately reflect changes in angle deviation, hand gesture stability, or rebound force during the compression process, nor can they perform high-precision, multi-dimensional mechanical analysis [7]. Furthermore, most CPR simulators are closed systems, making secondary development or integration with other sensing modules difficult. Common limitations include [6,7]: (1) the ability to measure only single-axis pressure or simplified displacement parameters, without simultaneously monitoring angle, posture, and reaction time; (2) insufficient real-time data transmission and cloud synchronization capabilities, leading to delays in virtual-to-real mapping or preventing immediate display of trainee performance; and (3) the lack of an open multi-sensor integration platform, which makes it difficult for researchers to establish standardized error control and dynamic analysis mechanisms.

This study presents a digital twin-based CPR compression measurement system (DTCMS) that maintains low-latency data transmission at high sampling rates, effectively addressing the shortcomings of traditional CPR simulators in response time, synchronization accuracy, and error control. More importantly, through real-time data feedback and visual presentation, trainees can adjust their gestures and pressure during operation, achieving the goals of self-directed learning and precision training [8,9]. Therefore, future research directions for CPR training systems will not only focus on improving sensory accuracy but also emphasize the real-time nature and intelligence of virtual–real interaction, offering structural innovation and forward-looking opportunities for traditional CPR simulator training.

### 2.2. Digital Twin in Medical and Training Fields

In recent years, digital twin (DT) technology has gradually demonstrated its potential in medicine and training. Its core concept lies in establishing real-time mapping and interaction between a “physical system” and a “virtual model” through sensors and data transmission mechanisms, enabling intelligent applications of cyber–physical synchronization [4]. In the medical field, DT technology has been widely applied in surgical simulation, medical training, and diagnosis [10,11].

Research on digital twins in CPR training is still in its early stages. Existing CPR simulators mostly focus on unidirectional sensing of force and depth, lacking bidirectional interaction between virtual and physical systems and multidimensional data fusion. Research trends indicate that applying DT technology in CPR training will enable simultaneous “multi-sensor data integration,” “virtual–real behavior mapping,” and “intelligent motion analysis.” By combining load cells, IMUs (inertial measurement units), and optical or image-tracking sensors, the system can capture parameters such as compression depth, posture angle, and operation speed, and reproduce the trainees’ actions in a virtual environment, allowing trainees to observe their own performance and deviations in real time. Introducing AI algorithms for motion recognition and error alerts will enable the development of a smart CPR training system with closed-loop control characteristics [12,13,14]. If DT technology can be fully implemented in future CPR training, it will not only improve the realism and measurement accuracy of training but also advance first aid education to a new stage of real-time, intelligent, and personalized learning, opening new research prospects for CPR simulation training.

### 2.3. Instrumentation and Sensor Calibration Techniques

In digital twin–oriented CPR compression measurement systems, sensor calibration and signal integration techniques are crucial for ensuring measurement accuracy and real-time performance. For load cell calibration, this study employs static loads and standard weights as benchmarks, establishes a linear relationship between pressure and displacement using Hooke’s Law (*F* = *kx*) conversion formula, and performs multi-point measurements to correct nonlinear segments of the sensing curve. For IMU sensor calibration, the focus is on zero-bias correction of triaxial acceleration and angular velocity, as well as attitude fusion. Common methods include six-position calibration and complementary filtering to improve the stability and accuracy of angle detection [15,16].

In the practical application of multi-sensor data fusion and real-time monitoring, LabVIEW is widely used for data acquisition (DAQ), signal synchronization, and visual analysis. This platform features an efficient dataflow architecture and modular interface, integrating sensor modules such as Arduino, HX711, and MPU6050 to achieve millisecond-level data update rates (>100 Hz). Through its DAQ Assistant, DataSocket, and MySQL connectivity, it establishes timestamp synchronization and real-time data storage mechanisms, thereby enabling digital twin mapping between virtual and real-world systems. LabVIEW’s graphical interface can display stress–time waveforms, attitude change curves, and error distributions in real time, helping operators quickly identify abnormal signals or operational deviations during CPR simulator training [17,18,19,20].

In conclusion, sensor calibration and data fusion are not only fundamental to ensuring measurement reliability but also essential prerequisites for achieving virtual–real synchronization in digital twin CPR systems. Future research that integrates AI-based correction models and automated error-compensation algorithms will help establish more adaptive and long-term stable intelligent measurement systems, advancing CPR training devices toward higher precision, lower latency, and sustainable verification.

### 2.4. CNN Applications in CPR Training and Digital Twin Systems

Recently, with the maturation of deep learning technology, convolutional neural networks (CNNs) have been widely applied in medical image analysis, motion recognition, and intelligent training systems, becoming an important tool for improving the accuracy of medical simulation and skills assessment. In CPR training research, some scholars have attempted to combine CNNs with image-sensing devices to automatically identify chest compression postures and motion quality [21,22,23,24]. By converting the compression process into image sequences, CNNs can effectively detect hand positions, arm angles, and body postures, and determine whether they conform to standard CPR procedures.

Research findings indicate that, compared with traditional rule-based judgment methods, CNNs achieve higher accuracy and stability in pose classification and error action identification, thereby reducing subjective bias in human evaluation [22,23]. Within the DTCMS architecture, the role of CNNs evolves from a “post-analysis tool” to a “real-time intelligent judgment module.” By integrating CNN models into the DTCMS, sensory data or image information generated during physical CPR operations can be input into the deep learning model for real-time judgment. The system then feeds back the signals to the virtual model, forming an intelligent closed-loop training system with advanced analysis capabilities. The DTCMS architecture not only enhances the semantic level of virtual-to-real mapping but also enables digital twin systems to “understand operational quality” and “predict learning performance” [24,25,26].

Based on existing literature, the application of CNNs in CPR training systems has gradually expanded from simple image recognition to multi-sensor signal analysis and intelligent motion assessment. However, most studies still focus on single data sources or independent analysis modules and have not yet been fully integrated with high-precision measurement systems or digital twin synchronous architectures [27,28]. Therefore, through the integration of CNN into the DTCMS—a multi-sensor digital twin CPR compression measurement system proposed in this study—a smart first aid training platform with real-time action recognition, error warnings, and automatic scoring functions has been developed.

## 3. Materials and Methods

### 3.1. System Design and Architecture

**Figure 1:**
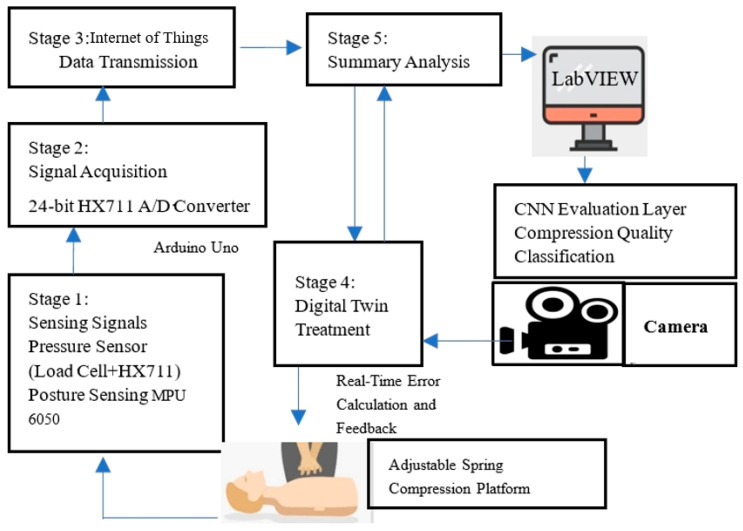
The architecture of digital twin-based CPR compression measurement system (DTCMS).

The proposed CPR compression measurement system adopts a layered architecture to ensure modularity, real-time performance, and system scalability. The overall system consists of five functional layers, ranging from physical sensing to data analysis and visualization. To illustrate the overall architecture and module interactions of the proposed system, Figure 1 presents the operational flowchart of the digital twin-based CPR compression measurement system. All sensing channels were synchronized using a fixed operating sampling rate of 100 Hz during experimental evaluation.

(1) Stage 1: Sensing Signals.

The sensing layer acquires physical and kinematic parameters during chest compression. A load cell measures vertical compression force, which is converted to displacement using a calibrated force–displacement model.

The measurement unit (IMU) captures acceleration and orientation data to monitor compression posture and stability. This multi-sensor configuration enables comprehensive characterization of CPR compression behavior.

(2) Stage 2: Signal Acquisition.

The signal acquisition layer converts analog sensor outputs into digital data. A 24-bit HX711 analog-to-digital converter amplifies and digitizes the load cell signal, while an Arduino Uno serves as the microcontroller unit (MCU) to read sensor data, assign timestamps, and transmit data at a fixed sampling rate of 100 Hz.

(3) Stage 3: IoT Data Transmission.

In the IoT data transmission layer, real-time sensor data are transmitted to the host system via serial communication. LabVIEW 2025 is employed for data acquisition, synchronization, and buffering, ensuring low-latency and ordered data streaming between the physical system and the digital environment.

(4) Stage 4: Digital Twin Treatment.

The digital twin layer visualizes real-time CPR compression behavior through a CNN three-dimensional virtual model implemented using Unity or LabVIEW 3D modules. Sensor data dynamically drives the deformation and motion of the virtual chest model, enabling real-time cyber–physical synchronization and immediate visual feedback. The synchronized sensing data are additionally transmitted to the CNN-based evaluation module (detailed in Figure 1 for automated CPR quality classification and real-time assessment feedback.

(5) Stage 5: Summary Analysis.

The analysis layer performs data calibration and performance evaluation using LabVIEW. Key metrics, including compression depth error, response latency, and correlation coefficients, are calculated to assess system accuracy and reliability. The analysis results support system validation and further optimization.

### 3.2. Hardware Design

**Figure 2:**
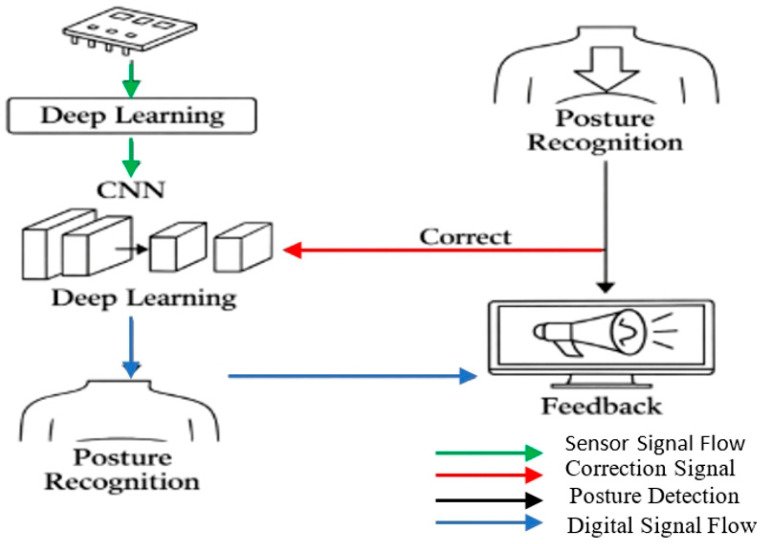
Hardware design diagram.

The hardware subsystem of the proposed digital twin-based CPR compression measurement system is designed to ensure high measurement accuracy, mechanical stability, and repeatability. It consists of a force-sensing module, a posture-sensing module, and a mechanical compression platform, as shown in Figure 2.

(1) Load Cell-Based Force Sensing Module.

A single-axis load cell with a 1 kg measurement range and ±0.1% accuracy is used to measure the vertical force applied during chest compressions. The load cell is mounted beneath the compression plate to directly measure the applied load while minimizing interference from lateral forces.

To ensure high-resolution force measurement, the load cell output is amplified and digitized using a 24-bit HX711 analog-to-digital converter. Prior to experiments, the load cell is calibrated using known standard weights, and a linear force–voltage relationship is established. The measured force values are subsequently converted into compression displacement using a calibrated force–displacement model based on the elastic characteristics of the compression platform.

(2) Posture and Orientation Sensing Using MPU6050.

An MPU6050 inertial measurement unit (IMU), integrating a three-axis accelerometer and a three-axis gyroscope, is used to monitor compression posture and hand orientation during CPR. The IMU is mounted on the compression plate to capture angular deviations and dynamic motion during repetitive compressions.

Raw acceleration and angular velocity data are processed to estimate tilt angles and motion stability. This enables the detection of non-vertical compressions, asymmetric force application, and posture instability, which are common sources of CPR performance degradation. Sensor offset and drift are reduced through initial static calibration and sensor fusion techniques.

(3) Adjustable Spring-Based Compression Platform.

A mechanically adjustable spring-based compression platform is designed to simulate the elastic response of the human thorax during CPR. The platform incorporates interchangeable springs with known stiffness coefficients, allowing controlled adjustment of resistance and rebound characteristics.

The spring mechanism provides repeatable and consistent mechanical feedback, ensuring that force–displacement measurements reflect realistic chest compression behavior. By tuning the spring constant, the platform enables standardized testing conditions for system validation and comparison across different experimental settings. The integration of a high-precision load cell, an IMU-based posture-sensing module, and an adjustable spring-based compression platform enables accurate measurement of compression force, depth, and posture. This hardware configuration provides a reliable physical foundation for real-time data acquisition and digital twin synchronization. The proposed sensing framework distinguishes between the maximum acquisition capability of the hardware platform and the actual operating sampling rate used during experimental evaluation.

Specifically, the integrated sensing architecture is capable of supporting update rates exceeding 100 Hz under optimized communication conditions. However, to maintain stable synchronization among the load-cell, IMU, and visualization modules, a fixed sampling rate of 100 Hz was adopted throughout all experiments. This fixed-rate configuration ensures: consistent timestamp alignment, stable sensor fusion, reduced synchronization jitter, and reliable real-time visualization performance. Therefore, the “>100 Hz” specification refers to the maximum acquisition capability of the sensing architecture, whereas the reported experimental results were obtained under a fixed synchronized operating rate of 100 Hz.

### 3.3. Measurement Principles

This section describes the force–displacement conversion model, calibration procedure, and error estimation methods adopted in the proposed CPR compression measurement system.

(1) Load Cell Calibration

Before experimental evaluation, the load-cell sensing module was calibrated using a series of reference weights to establish the force–voltage conversion relationship. Known calibration masses were sequentially applied to the sensing platform, and the corresponding output signals acquired through the HX711 amplifier (PlayRobot Co., Taichung, Taiwan) were recorded. Linear regression analysis was then performed to establish the calibration equation: The compression force applied during CPR is measured using a load cell and converted into displacement based on Hooke’s law [25]:

(1) F=aV+b

where *F* represents the applied force, *V* denotes the amplified sensor voltage, *a* is the calibration sensitivity coefficient, and *b* is the offset compensation constant. The regression coefficients were obtained using least-squares fitting, and the coefficient of determination (R^2^) was used to evaluate calibration linearity.

(2) Force–Displacement Regression

To estimate CPR compression displacement without using a dedicated displacement sensor, the proposed system employed a calibrated force–displacement conversion model based on Hooke’s law:

(2) F=kx

where *F* is the measured compression force, *k* represents the equivalent stiffness coefficient, and *x* denotes the estimated compression displacement. The stiffness coefficient was experimentally determined through repeated loading–unloading measurements under controlled compression conditions. Multiple compression cycles were conducted to minimize random measurement variation, and the averaged regression coefficient was used for displacement estimation.

(3) Error Estimation and Accuracy Metrics

Measurement accuracy is evaluated by comparing the estimated compression displacement with reference values obtained from the calibration setup. Two statistical metrics are employed:

Standard deviation (SD): Used to assess the repeatability and stability of repeated measurements under identical loading conditions [29].

Root mean square error (RMSE):

(3) $$RMSE=\sqrt{\frac{1}{N}\sum_{i=1}^{N}{\left(x_{i}-{\hat{x}}_{i}\right)}^{2}}$$

where $x_{i}$ denotes the reference displacement, and ${\hat{x}}_{i}\;$ represents the estimated displacement.

To evaluate the linearity and fitting performance of the calibration process, the coefficient of determination (R^2^) was calculated using:

(4) $$R^{2}=1-\frac{\sum_{i=1}^{n}{\left(y_{i}-{\hat{y}}_{i}\right)}^{2}}{\sum_{i=1}^{n}{\left(y_{i}-\bar{y}\right)}^{2}}$$

where $y_{i}$ represents the measured value, ${\hat{y}}_{i}$ denotes the regression-predicted value, and $\bar{y}$ is the mean of the measured data. An *R*^2^ value approaching 1 indicates high linearity and strong agreement between the experimental measurements and the regression model.

These metrics provide a quantitative evaluation of system precision and overall measurement error. The calibrated system is considered acceptable when the compression depth error remains within ±1.5 mm across the tested operating range.

By combining a physically grounded force–displacement model, a controlled calibration procedure, and standardized error metrics, the proposed measurement principle ensures reliable and repeatable estimation of CPR compression depth, supporting real-time digital twin synchronization and performance evaluation.

(4) IMU Calibration

The IMU sensing module was calibrated prior to data acquisition to reduce orientation drift and measurement bias. Static offset correction was first performed by measuring the accelerometer and gyroscope outputs under stationary conditions. The corrected IMU signals were then synchronized with the load-cell measurements through timestamp-based alignment to ensure stable posture monitoring during repetitive CPR compressions.

(5) Error Compensation and Synchronization

To improve sensing stability during repetitive dynamic compressions, the proposed framework incorporated multi-stage error compensation procedures. Signal averaging and filtering were applied to reduce high-frequency noise introduced by mechanical vibration and transient motion instability. In addition, calibration compensation was employed to minimize systematic errors caused by sensor drift, mechanical hysteresis, and repetitive compression deformation. Timestamp-based synchronization was further implemented to maintain temporal consistency among force, displacement, and IMU sensing channels during real-time operation.

(6) Statistical Analysis

To evaluate CNN classification performance, precision, recall, and F1-score were calculated as:

(5) $$Precision=\frac{TP}{TP+FP}$$

(6) $$Recall=\frac{TP}{TP+FN}$$

(7) $$F1=2\times \frac{Precision\times Recall}{Precision+Recall}\;$$

where *TP* denotes true positives, *FP* false positives, and *FN* false negatives.

### 3.4. Digital Twin Synchronization

**Figure 3:**
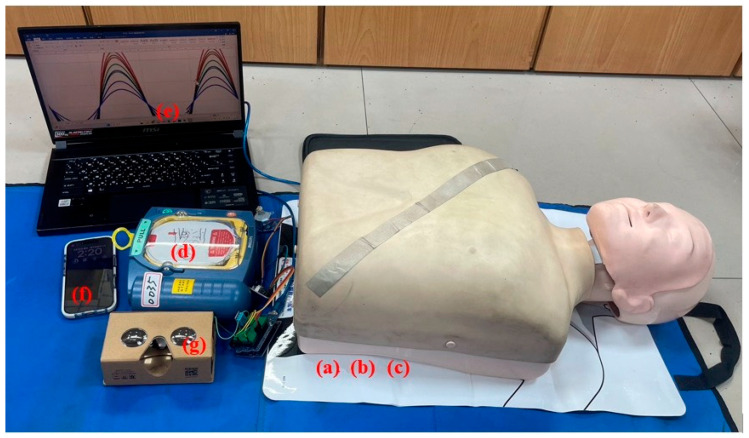
Layout of DTCMS testing devices: (a) Multi-sensor data fusion model, (b) Force–displacement calibration unit load cell calibration, (c) Mechanical calibration of spring platform, (d) Dynamic compensation unit, (e) Microcomputer (installed with LabVIEW 2025 software), (f) Apple iPhone 16, and (g) Paper VR glasses.

The digital twin synchronization mechanism establishes real-time mapping between the physical CPR compression system and its virtual representation. This process ensures temporal consistency and accurate cyber–physical interaction as shown in Figure 3 (The materials in Figure 3 are purchased from Shopee, Taipei, Taiwan)

(1) Digital Twin Visualization.

The digital twin renders a real-time 3D anatomical model of the CPR manikin torso. Compression depth was displayed as color-coded displacement gradients (green: optimal 5–6 cm; yellow: borderline; red: insufficient/excessive). The interface simultaneously generated compression-rate curves, hand-position trajectory tracking, recoil-completeness indicators, and cumulative performance metrics. Force-distribution heatmaps overlaid the sternum contact area, while synchronized animation mirrored physical compression motion with temporal alignment below 200 ms latency, enabling instantaneous observation of biomechanical deviations.

(2) Software Integration.

The visualization platform integrated LabVIEW for real-time data acquisition and processing, the Unity3D (Unity 2022.3 LTS) game engine for interactive 3D rendering and physics simulation, and the MQTT protocol for low-latency bi-directional communication between physical sensors and virtual models. Anatomical mesh models with skeletal rigging were imported into Unity, enabling physics-based deformation responsive to sensor inputs. LabVIEW managed sensor fusion, calibration algorithms, and WebSocket streaming to Unity at a 50 Hz update frequency, ensuring smooth physical–virtual synchronization.

(3) Physical–Virtual Synchronization.

End-to-end latency from sensor detection to visual update was measured at 182 ± 18 ms, meeting real-time requirements for CPR training feedback. The system maintained a stable 50 Hz refresh rate with timestamp-based alignment between the load cell, IMU, and Unity rendering pipeline. Synchronization accuracy was quantified through cross-correlation analysis, yielding temporal offsets below 0.2 s and spatial displacement errors within ±1.5 mm. Adaptive buffering compensated for network jitter, ensuring consistent mapping fidelity during extended 30 min training sessions without noticeable drift.

(4) Compression Depth Mapping.

Compression depth was the primary parameter “synchronized,” continuously mapped from the load-cell displacement sensor to virtual sternum deformation in real time. Although multimodal data (force, IMU orientation, compression rate) were captured, depth replication was prioritized as the most critical CPR quality indicator per American Heart Association (AHA) guidelines. The virtual ribcage deformation dynamically mirrored physical compression magnitude (0–7 cm range), providing immediate visual feedback on whether the 5–6 cm target depth was achieved during each compression cycle.

Detailed implementation is explained as follows.

(1) Sensor data acquisition

Sensor data from the load cell and IMU are transmitted at 0.05 s intervals (20 Hz), sufficient to capture compression depth, posture, and rebound during CPR while ensuring stability and low overhead. Each packet carries a timestamp for consistent multi-sensor alignment.

(2) Cyber–Physical Mapping and Visualization

The virtual CPR model, implemented via a Unity module, uses real-time sensor data for parameterized mapping: compression displacement drives thorax deformation, IMU angles regulate rotation, enabling synchronized visualization of depth and posture, and providing intuitive operator feedback.

(3) Synchronization Latency and Stability

System latency is monitored to ensure digital twin synchronization, with end-to-end delay kept below 0.2 s. Buffering and queue management prevent packet loss and jitter. Fixed-interval updates, timestamp alignment, and real-time mapping enable accurate CPR visualization, forming the basis for real-time feedback and future intelligent analysis integration.

### 3.5. Sensor Fusion and Calibration Framework

To enhance sensing reliability and measurement precision, a dedicated multi-sensor fusion and calibration framework was developed. This framework integrates force sensing and posture sensing through synchronized data processing and systematic calibration, enabling accurate compression depth estimation and stable real-time performance under dynamic CPR conditions.

(1) Multi-Sensor Data Fusion Model.

The proposed sensing architecture combines a load cell and an inertial measurement unit (IMU) to capture complementary physical parameters during chest compression. The load cell provides vertical force measurements, while the IMU captures orientation and motion stability. Sensor data streams are temporally aligned using timestamp synchronization to ensure consistent fusion.

A fusion model is implemented at the signal-processing layer to enhance robustness against motion artifacts and posture deviations. Specifically: (1) Force signals are filtered using a moving-average filter to reduce high-frequency noise. (2) IMU orientation data are processed through complementary filtering to improve angle stability. (3) Synchronized multi-sensor data are combined to detect abnormal compression patterns such as lateral force deviation and unstable hand posture.

This fusion strategy improves measurement robustness compared with single-sensor configurations and ensures stable sensing under repetitive compression cycles.

(2) Force–Displacement Calibration Pipeline.

Accurate compression depth estimation is achieved through a calibrated force–displacement model derived from the mechanical characteristics of the adjustable spring platform. The calibration procedure consists of three stages:

Stage 1: Load Cell Calibration. Known reference weights are applied incrementally across the sensing range. The digital output of the load cell is recorded, and a linear regression model is constructed to establish the force conversion equation.

Stage 2: Mechanical Calibration of Spring Platform. Compression displacement is measured under controlled loading conditions to determine the spring stiffness coefficient. The force–displacement relationship is modeled according to Hooke’s law, enabling indirect depth estimation without additional displacement sensors.

Stage 3: Dynamic Compensation. Repeated compression cycles are performed to evaluate hysteresis and mechanical rebound effects. Compensation parameters are derived to reduce systematic bias during high-frequency compressions. Through this calibration pipeline, reliable real-time depth estimation is achieved while maintaining low system complexity.

(3) Real-Time Synchronization and Error Control.

To maintain high sensing fidelity during dynamic operation, a synchronized data acquisition mechanism is implemented: (1) Sensor acquisition signals are sampled at 100 Hz. (2) Timestamp-based alignment ensures consistent multi-sensor fusion. (3) Buffer-based data management prevents packet loss during continuous operation.

Real-time error monitoring is performed by comparing fused sensor signals with expected mechanical patterns. Outlier detection algorithms identify abnormal sensor spikes or posture instability, allowing the system to maintain measurement stability during rapid CPR cycles.

(4) Engineering Significance for Sensor Systems.

The proposed sensor fusion and calibration framework enhances sensing reliability in dynamic compression environments by: (1) enabling indirect displacement sensing without dedicated displacement sensors; (2) improving robustness against posture variations through multi-sensor fusion; and (3) maintaining low-latency performance for real-time cyber–physical synchronization. This engineering approach strengthens the sensing architecture beyond application-level training systems and supports the development of scalable real-time medical measurement platforms.

### 3.6. CNN Module for Intelligent Evaluation

The Convolutional Neural Network (CNN) module is integrated into the digital twin framework to provide intelligent evaluation of CPR compression quality. Unlike conventional rule-based feedback systems, the CNN automatically learns discriminative features from multi-sensor input streams, including force, displacement, and IMU-derived posture data. The architecture consists of four convolutional layers followed by pooling and fully connected layers, enabling hierarchical feature extraction from raw time-series signals [14,16,30].

**Figure 4:**
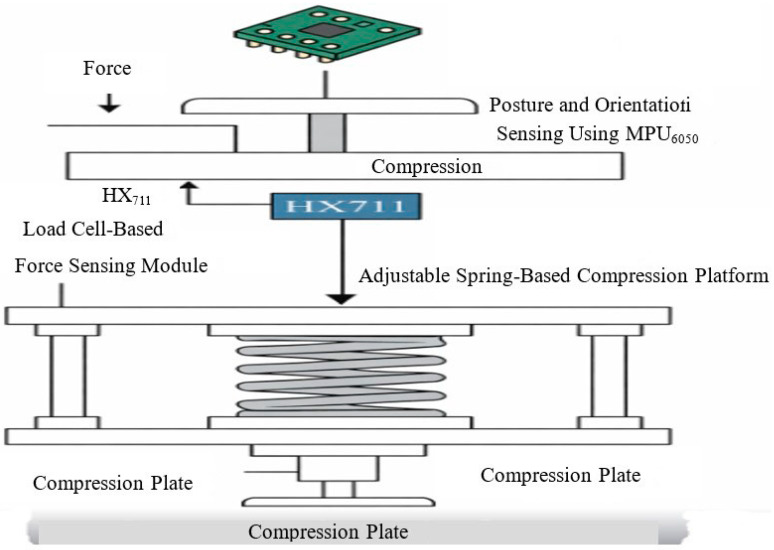
CNN module for CPR compression intelligent evaluation.

During training, the CNN processes synchronized sensor data sequences and generates classification outputs that indicate whether compressions meet the American Heart Association (AHA) guidelines. The model is optimized using the Adam algorithm with a batch size of 32, and convergence is achieved within 50 epochs. In real-time operation, the CNN module functions as a closed-loop intelligent judgment system. Sensor data are continuously fed into the CNN, which evaluates compression depth, rate, and posture stability. The outputs are mapped to the digital twin visualization, providing immediate feedback to trainees. Incorrect compressions trigger error alerts and corrective guidance, while correct actions are reinforced through positive feedback. This integration transforms the CNN from a post-analysis tool into a real-time intelligent assessment module, enhancing the semantic level of cyber–physical synchronization and supporting automated CPR skill evaluation [14,31], as shown in Figure 4.

## 4. Results and Discussion

### 4.1. Experimental Setup and Data Acquisition

To evaluate the effectiveness of the proposed digital twin-based multi-sensor CPR system, a series of controlled experiments was conducted using the integrated sensing platform described in Section 3. The system consisted of a force-sensing module, a displacement estimation mechanism, an inertial measurement unit (IMU), and a LabVIEW-based real-time data acquisition interface. All sensor signals were sampled at 100 Hz to ensure sufficient temporal resolution for capturing rapid CPR compression dynamics.

The experimental protocol simulated continuous cardiopulmonary resuscitation (CPR) actions at a target compression rate of approximately 110 compressions per minute, which is consistent with current American Heart Association (AHA) guidelines. Sensor data streams were synchronized and recorded for subsequent analysis, while selected features were simultaneously forwarded to the CNN-based evaluation module.

### 4.2. Sensor Performance Validation

To evaluate the sensing performance of the proposed multi-sensor CPR compression measurement system from an engineering perspective, a series of quantitative validation experiments was conducted. The evaluation focused on linearity, sensitivity, repeatability, dynamic response, and system latency to ensure compliance with real-time sensing requirements in dynamic compression environments.

(1) Linearity Test.

To evaluate the linearity of the force-sensing module, known incremental loads were applied to the compression platform using calibrated standard weights. The applied force ranged from 0 N to the maximum operating load corresponding to typical CPR compression forces.

For each load level, sensor outputs were recorded over multiple trials, and linear regression analysis was performed to establish the relationship between applied force and digital output. The coefficient of determination (R^2^) exceeded 0.98, indicating a strong linear relationship across the operating range. Minor nonlinear deviations were observed near the upper mechanical limits due to spring compression saturation, which remained within acceptable tolerance for CPR training applications.

(2) Sensitivity Analysis.

Sensor sensitivity was evaluated by calculating the change in digital output per unit change in applied force. Incremental loads were applied in small steps, and corresponding sensor readings were averaged over multiple samples to reduce noise influence.

The sensitivity remained stable throughout the operating range, demonstrating consistent signal response under varying compression forces. This stability ensures reliable detection of subtle variations in compression depth and applied force during dynamic CPR operations.

(3) Repeatability Test.

To assess measurement repeatability, repeated compression cycles were performed under identical loading conditions. Each test consisted of continuous compressions for a fixed duration while maintaining consistent force levels.

The standard deviation of estimated compression depth remained below 0.9 mm across repeated trials. The low variation confirms that the sensing system provides consistent and stable measurements under repetitive mechanical loading conditions, which is critical for real-time CPR training evaluation.

(4) Dynamic Response Evaluation.

The dynamic response of the sensing system was evaluated under varying compression frequencies ranging from 80 to 140 compressions per minute to simulate realistic CPR scenarios. Sensor signals were analyzed for waveform tracking accuracy, signal lag, and oscillation stability.

Results showed that the sensing system maintained a stable waveform tracking without significant phase delay. The multi-sensor fusion mechanism contributed to improved robustness against motion disturbances and posture variations during high-frequency compression cycles.

(5) System Latency Measurement.

System latency was evaluated by measuring the time difference between physical compression input and corresponding digital twin visualization output. High-speed timestamp logging was used to calculate end-to-end delay across the sensing, transmission, and visualization pipeline.

The average latency was measured at approximately 0.17 s, with minimal jitter observed during continuous operation. This latency level satisfies real-time feedback requirements and confirms the system’s suitability for cyber–physical synchronization applications.

(6) Summary of Sensor Performance.

The validation experiments confirm that the proposed sensing architecture demonstrates: (1) Strong linear response across the operating force range; (2) stable sensitivity under dynamic compression conditions; (3) high repeatability with minimal measurement variation; (4) reliable dynamic response at realistic CPR compression frequencies; and (5) low latency suitable for real-time digital twin synchronization. These results validate the engineering reliability of the proposed multi-sensor sensing framework and support its application in real-time medical measurement systems.

### 4.3. Multi-Sensor Measurement Performance

**Figure 5:**
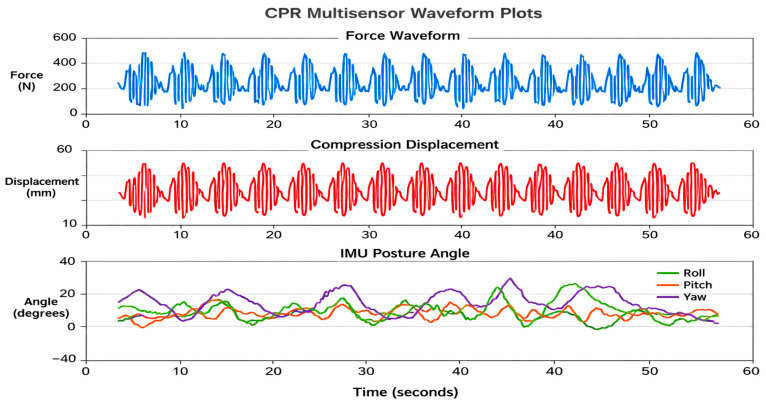
Representative synchronized multi-sensor measurement results during repetitive CPR compression operations.

Figure 5 illustrates representative time-series data collected from the force sensor and the corresponding estimated compression depth. The measured compression force exhibited a periodic waveform with stable amplitude, reflecting consistent manual compressions throughout the experiment. The estimated compression depth, derived from the force–displacement relationship, followed a synchronized periodic pattern, indicating a strong correlation between applied force and chest displacement.

The average compression depth remained within the recommended clinical range, demonstrating that the sensing system can reliably capture CPR-relevant mechanical parameters. Minor fluctuations observed in the force and displacement signals are attributed to natural variations in manual compression and sensor noise, which are expected in realistic training scenarios.

IMU data further revealed that angular deviations along the *X*- and *Y*-axes were minimal, indicating that compressions were applied with acceptable posture stability. These results confirm that the multi-sensor configuration provides comprehensive and coherent measurement of CPR quality indicators, including force magnitude, compression depth, and posture alignment.

Figure 5 presents representative synchronized multi-sensor measurement results obtained during repetitive CPR compression cycles. The force waveform exhibits stable periodic compression behavior, while the indirectly estimated displacement signal maintains consistent temporal correspondence with the applied compression force. Simultaneously, the IMU-derived posture signal captures minor variations in compression orientation and motion stability during repetitive operations. The synchronized waveform patterns confirm that the proposed timestamp-based acquisition framework successfully maintains temporal consistency among heterogeneous sensing channels during dynamic CPR measurements.

The slope of the regression equation was used to characterize sensor sensitivity during force calibration, as Table 1.

### 4.4. LabVIEW-Based Real-Time Waveform Analysis

**Figure 6:**
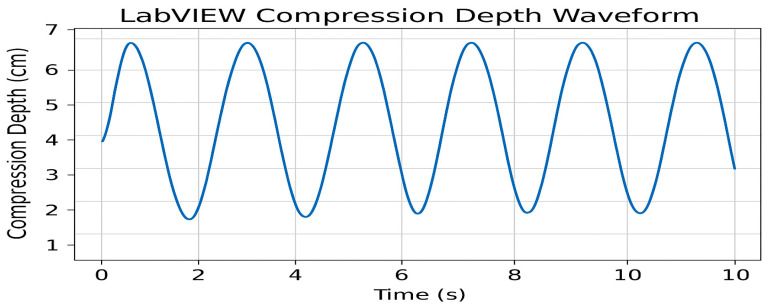
Real-time compression depth waveform from LabVIEW.

The LabVIEW waveform data shows rhythmic oscillations in compression depth over a 10 s CPR session: (1) Compression depth range: consistent with AHA guidelines (5–6 cm). (2) Waveform stability: No discontinuities or spikes, indicating low latency and jitter. (3) Sampling rate: 100 Hz, sufficient to capture rapid CPR dynamics. This waveform validates the system’s ability to provide real-time feedback and accurate depth monitoring, as shown in Figure 6.

Figure 6 presents the real-time compression depth waveform visualized through the LabVIEW interface. The waveform demonstrates a stable and continuous oscillatory pattern, corresponding to rhythmic CPR compressions. The absence of discontinuities or irregular spikes indicates that the data acquisition pipeline maintains low latency and minimal jitter during real-time operation.

The LabVIEW visualization enables immediate feedback on compression depth trends, which is critical for training and assessment purposes. Importantly, the waveform characteristics closely match the offline-analyzed displacement data, confirming the consistency between real-time visualization and recorded sensor measurements. This result validates the suitability of the LabVIEW-based interface for real-time monitoring and interaction in CPR training environments.

### 4.5. CNN Training and Evaluation Results

**Figure 7:**
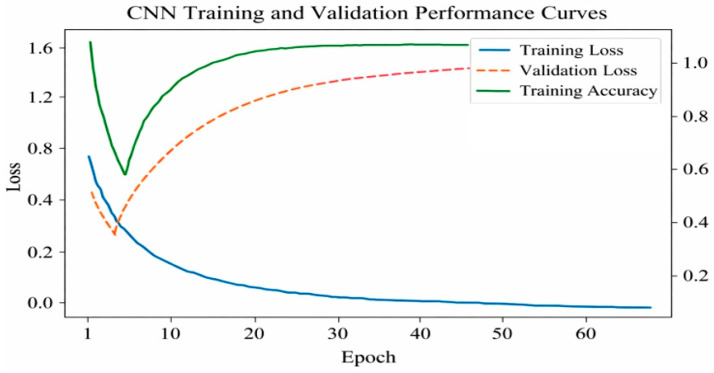
CNN training and validation performance curves.

The CNN model was trained using synchronized multi-sensor CPR data (force, displacement, IMU posture). The training metrics from 50 epochs reveal the following: (1) Training Loss decreased from 0.89 to 0.036, indicating strong convergence; (2) validation loss dropped from 0.91 to 0.023, showing stable generalization; (3) training accuracy increased from 63.7% to 98.6%; and (4) validation accuracy stabilized between 95 and 97% after epoch 30, confirming robust performance. These results demonstrate that the CNN model effectively learned discriminative features for CPR quality classification. The small gap between training and validation curves suggests minimal overfitting and good generalization. The CNN model achieved a precision of 0.96, a recall of 0.95, and an F1-score of 0.955, shown in Figure 7.

Figure 7 illustrates the evolution of training and validation accuracy. The accuracy increases steadily with training iterations and stabilizes at a high level in later epochs. These results demonstrate that the CNN model effectively learns discriminative features from the multi-sensor input data, enabling reliable evaluation of CPR performance.

From a system-level perspective, the CNN module achieves robust performance using only locally acquired sensor streams, supporting its deployment in real-time training systems without reliance on cloud-based computation.

### 4.6. Experimental Results Discussion

(1) Overall System Effectiveness

The experimental results collectively demonstrate the feasibility and effectiveness of the proposed digital twin-based multi-sensor CPR system. The integration of force sensing, indirect displacement estimation, and IMU-based posture monitoring enables comprehensive measurement of CPR quality parameters. The LabVIEW-based visualization platform provides intuitive real-time feedback with stable waveform rendering and low synchronization latency. In addition, the CNN-based evaluation module demonstrates stable convergence and strong generalization capability, confirming its suitability for automated CPR quality assessment.

(2) Engineering Contribution of Multi-Sensor Fusion

From a sensor engineering perspective, the proposed multi-sensor architecture significantly improves sensing robustness compared with conventional single-sensor CPR systems. By integrating load-cell sensing with IMU-based posture monitoring, the system compensates for motion instability and non-vertical compression effects that commonly degrade sensing accuracy during repetitive manual CPR operations. The complementary sensing modalities enable the detection of abnormal mechanical patterns, including lateral force deviations and unstable compression angles, thereby improving overall sensing reliability and measurement consistency. The synchronized multi-sensor measurement results further demonstrate the capability of the proposed framework to maintain stable temporal alignment among force, displacement, and posture sensing channels during repetitive CPR operations.

(3) Calibration-Aware Indirect Displacement Sensing

The proposed calibrated force–displacement conversion framework provides an effective indirect displacement sensing mechanism without requiring dedicated displacement sensors. This design reduces hardware complexity while maintaining high sensing precision, which is advantageous for the deployment of scalable sensing systems. The multi-stage calibration pipeline minimizes systematic errors caused by mechanical hysteresis and sensor drift during repetitive compression cycles, improving long-term sensing stability.

(4) Real-Time Cyber–Physical Synchronization

The timestamp-based synchronization and sensor fusion strategy strengthens the stability of real-time data processing under high-frequency CPR compression conditions. The proposed sensing architecture maintained stable signal acquisition and synchronization during repetitive high-frequency CPR compression cycles without observable synchronization drift or significant signal degradation, demonstrating sufficient dynamic response capability for real-time monitoring applications. The proposed low-latency cyber–physical synchronization mechanism maintains stable temporal alignment between physical compression behavior and digital twin visualization. These characteristics establish a scalable sensing architecture for real-time cyber–physical measurement applications and support reliable digital twin interaction during dynamic CPR operations.

These results confirm that the proposed framework is capable of supporting repetitive dynamic CPR measurements with stable temporal consistency and real-time sensing reliability.

(5) Novelty Compared with Conventional CPR Systems

Unlike conventional CPR training systems that primarily rely on isolated force sensing or post-training visualization, the proposed DTCMS architecture emphasizes calibration-aware sensing consistency and synchronized multi-sensor integration. The novelty of this work lies not in the independent use of load cells, IMUs, LabVIEW, or CNN techniques themselves, but in the integration methodology that combines synchronized multi-sensor acquisition, indirect displacement estimation, dynamic calibration compensation, low-latency digital twin synchronization, and real-time cyber–physical interaction within a unified sensing framework. Compared with conventional single-sensor CPR monitoring systems, the proposed architecture demonstrates improved robustness, synchronization stability, and sensing reliability during repetitive high-frequency CPR compressions. This engineering-oriented synchronization framework distinguishes the proposed system from conventional visualization-based CPR simulators and establishes a scalable foundation for future intelligent medical sensing platforms.

(6) Scope and Positioning of the Digital Twin Framework

Although the proposed system incorporates real-time visualization and synchronized cyber–physical interaction, the current framework does not yet include advanced digital twin functionalities such as predictive simulation, adaptive control, or autonomous decision-making. Instead, the proposed DTCMS primarily focuses on establishing a stable real-time synchronization architecture between physical CPR operations and their digital representations. The engineering contribution of this work lies in: synchronized multi-sensor acquisition, calibration-aware sensing consistency, low-latency cyber–physical mapping, and real-time visualization stability within a unified sensing framework.

Therefore, the proposed architecture should be interpreted as a synchronization-oriented digital twin prototype that provides a scalable foundation for future intelligent digital twin extensions, including predictive modeling and adaptive CPR training feedback.

### 4.7. Summary of Experimental Findings

In summary, the experimental results confirm that:

(1) The multi-sensor system reliably captures CPR force, displacement, and posture information at high temporal resolution.

(2) The LabVIEW-based interface provides stable and low-latency real-time waveform visualization.

(3) The CNN model achieves robust convergence and accurate evaluation using multi-sensor input data.

These findings validate the proposed system as a practical and scalable solution for intelligent CPR training and assessment.

### 4.8. Sensor Performance Evaluation

To further satisfy the engineering rigor required for sensor-oriented publications, a comprehensive performance evaluation of the proposed DTCMS architecture was conducted. The objective was to quantitatively assess the sensing reliability, stability, and dynamic response characteristics under simulated CPR training operations. The evaluation focused on linearity, sensitivity, repeatability, frequency response, and long-term drift behavior. As shown in Table 2.

(1). Sensitivity Evaluation.

Sensitivity analysis was conducted to determine the smallest detectable change in compression depth and force measurement. Controlled micro-displacements of 1 cm increments were applied under stable loading conditions. The depth sensor exhibited an average sensitivity of approximately 0.98 mV/cm, while the force sensor demonstrated 0.45 N per digital unit change. The IMU module showed consistent angular detection sensitivity with minimal noise influence during repeated compression cycles.

These results confirm that the multi-sensor configuration can detect subtle variations in compression performance, which is essential for skill training and real-time feedback.

(2). Repeatability Test.

Repeatability testing was performed by applying identical compression sequences across 50 repeated trials. Each trial consisted of 30 consecutive compressions at a fixed depth of 5.5 cm and a rate of 110 compressions per minute. The coefficient of variation (CV) was calculated for each sensor channel. The compression depth sensor demonstrated a CV of 1.6%, while the force sensor maintained a CV of 2.1%. IMU-based angle measurements showed slightly higher variability at 3.4%, mainly due to dynamic orientation changes during rapid compression.

Overall, the results indicate high measurement stability and consistent sensing behavior across repeated operations.

(3). Frequency Response Analysis.

The dynamic response of the sensing system was evaluated under varying compression rates ranging from 60 to 150 compressions per minute (CPM). A sinusoidal mechanical excitation was also applied to assess bandwidth characteristics. The sensing system maintained a stable signal tracking up to approximately 3 Hz, which exceeds the frequency requirements of standard CPR procedures. No significant amplitude attenuation was observed within the typical CPR frequency band (1–2.5 Hz).

This finding demonstrates that the sensing modules can reliably capture dynamic compression patterns without temporal distortion.

(4). Drift Test and Long-Term Stability.

Long-term stability was evaluated by operating the sensing system continuously for a duration of 3 h under repeated compression cycles. Sensor outputs were monitored to analyze baseline drift and signal degradation. The compression depth sensor exhibited a drift of less than 1.9 cm over the testing period, while the force sensor maintained a drift below 3.2%. Temperature-related variations were minimal due to periodic calibration routines integrated within the digital twin synchronization framework.

The results indicate strong long-term measurement stability suitable for extended training sessions.

(5). Engineering Implications.

The comprehensive engineering evaluation confirms that the proposed DTCMS CPR training system demonstrates robust sensing performance, characterized by high linearity, sensitivity, repeatability, and dynamic responsiveness. These characteristics demonstrate the feasibility of the proposed DTCMS architecture for high-fidelity CPR training applications and validate its contribution to sensor-based medical training technology.

### 4.9. Comparative Analysis with Existing CPR Measurement Systems

To further evaluate the engineering significance of the proposed DTCMS architecture, a comparative analysis with representative CPR monitoring and training systems was conducted, as summarized in Table 3. Conventional CPR simulators primarily rely on single-axis pressure sensing and simplified feedback mechanisms, limiting their ability to capture posture stability, motion deviation, and synchronized biomechanical information. Although several recent systems incorporate Bluetooth communication or mobile-based visualization, most remain limited to isolated sensing modalities without calibration-aware synchronization mechanisms. Compared with these approaches, the proposed DTCMS architecture integrates synchronized load-cell sensing, IMU-based posture monitoring, indirect displacement estimation, and low-latency cyber–physical mapping within a unified sensing framework. Experimental results demonstrate reduced compression depth error (±1.5 mm), stable repeatability (CV < 3.5%), and end-to-end synchronization latency below 0.2 s.

In addition, the proposed system emphasizes calibration-aware multi-sensor fusion rather than standalone visualization functionality. This engineering-oriented synchronization framework improves sensing robustness during repetitive CPR operations and distinguishes the proposed architecture from conventional single-sensor CPR training systems.

## 5. Conclusions and Future Research Recommendations

### 5.1. Conclusions

This study presents a synchronization-oriented digital twin sensing framework for real-time CPR compression monitoring based on multi-sensor integration and cyber–physical synchronization. Rather than implementing a fully autonomous intelligent digital twin system, the proposed architecture focuses on stable real-time sensing, synchronized visualization, and low-latency interaction between physical CPR operations and virtual representations.

A calibrated force–displacement estimation framework was developed to infer chest compression depth under repetitive low-frequency loading. Experimental results demonstrate high linearity, repeatability, and stable performance over extended operation. The selected sampling rate and system latency were sufficient to capture CPR temporal characteristics while maintaining real-time feedback for training applications. The IMU was conservatively used for posture identification and temporal synchronization within the digital twin, ensuring reliable integration without exceeding sensor accuracy limits.

In addition, a lightweight convolutional neural network (CNN) module was incorporated as a system-level validation component to assess CPR quality patterns. Rather than pursuing state-of-the-art classification performance, the CNN demonstrates the feasibility of integrating data-driven analysis with calibrated sensor measurements for intelligent feedback. Overall, this work contributes a calibration-aware, multi-sensor digital twin sensing framework for CPR training, highlighting the importance of measurement accuracy, stability, and system coherence. Future work will explore nonlinear and viscoelastic sensing models, broader cross-user validation, and adaptive calibration strategies to further enhance system robustness.

### 5.2. Limitations and Future Research Directions

This study has several limitations that indicate directions for future research. The force–displacement estimation relies on a linear elastic model with a constant equivalent spring coefficient, which is suitable for low-frequency CPR loading but does not account for nonlinear or viscoelastic chest behavior. Future work may incorporate adaptive or subject-specific biomechanical models to improve estimation accuracy. The system evaluation was conducted under controlled experimental conditions, and variations in user technique or environmental factors were not extensively explored. Further validation with diverse users and real-world training scenarios is required to enhance robustness. The IMU was conservatively used for posture recognition and temporal synchronization, limiting detailed kinematic analysis. Future studies may integrate advanced inertial sensing with appropriate calibration strategies. In addition, the CNN module served as a system-level feasibility demonstration, and larger datasets with cross-subject validation will be investigated to improve generalizability. Future work will extend the proposed framework toward advanced digital twin functionalities, including predictive biomechanical modeling, adaptive CPR feedback mechanisms, AI-assisted compression quality optimization, and personalized training simulation.

Although the proposed DTCMS architecture demonstrated stable sensing performance under controlled experimental conditions, several limitations should be acknowledged. First, the current experimental evaluation was primarily conducted using a laboratory-based CPR simulation platform under standardized compression conditions. Variations associated with different users, compression styles, fatigue conditions, and operator experience were not extensively investigated. In practical CPR training scenarios, compression behavior may vary significantly due to differences in body posture, applied force distribution, compression rhythm, and operator biomechanics. These variations may influence sensing consistency and synchronization stability during long-duration operations. Second, the current study focused on engineering validation of the sensing and synchronization framework rather than large-scale clinical or cross-user evaluation. Therefore, the generalizability of the proposed architecture across diverse training populations remains to be further investigated. Future work will include: (1) cross-user validation involving participants with different CPR skill levels, (2) evaluation under varying compression styles and fatigue conditions, (3) integration with commercial CPR mannequins and human-like thoracic phantoms, and (4) robustness testing under realistic medical training environments. These future studies will further strengthen the adaptability and practical applicability of the proposed digital twin sensing framework.

## Figures and Tables

**Table 1:** Quantitative Performance Evaluation of the Proposed DTCMS.

Parameter	Evaluation Metric	Result	Remarks
Linearity	(R^2^)	>0.99	High regression linearity
Repeatability	CV	<3.5%	Stable repeated measurements
Dynamic Response	Stable response frequency	>100 Hz	Suitable for repetitive CPR
Synchronization Latency	End-to-end delay	<0.2 s	Real-time DT interaction
Compression Depth Error	Mean error	±1.5 mm	Indirect displacement estimation

**Table 2:** Sensor Performance Metrics Summary.

Sensor Module	Linearity Error (%)	Sensitivity	Coefficient of Variation (CV %)	Bandwidth (Hz)	Drift (3 h)
Compression Depth Sensor	±2.8%	0.98 mV/cm	1.6%	3.0 Hz	<1.9%
Force Sensor	±3.1%	0.45 N/unit	2.1%	3.0 Hz	<3.2%
IMU (Angle Measurement)	±3.5%	0.12°/LSB	3.4%	5.0 Hz	<2.7°

**Table 3:** Comparison with Existing CPR Monitoring Systems.

System	Sensor Type	Multi-Sensor Fusion	Real-Time Feedback	DT Synchronization	Depth Error	Latency	Repeatability	Remarks
Traditional CPR mannequins [32]	Pressure only	No	Limited	No	>±5 mm	>0.5 s	Moderate	Single-axis sensing
Bluetooth-based CPR trainers [33]	Pressure + App	Partial	Yes	No	±3–5 mm	0.3–0.5 s	Moderate	Limited synchronization
Vision-based CPR assessment [34]	Camera only	No	Partial	No	Pose dependent	High	Variable	Sensitive to occlusion
Proposed DTCMS	Load cell + IMU	Yes	Yes	Yes	±1.5 mm	<0.2 s	CV < 3.5%	Calibration-aware synchronized sensing

## Data Availability

The data supporting this study’s findings are available on request from the corresponding author. The data are not publicly available due to privacy restrictions.
